# A description of *Echinorhynchus baeri* Kostylew, 1928 (Acanthocephala: Echinorhynchidae) from *Salmo trutta* in Turkey, with notes on synonymy, geographical origins, geological history, molecular profile, and X-ray microanalysis

**DOI:** 10.1051/parasite/2016067

**Published:** 2016-12-19

**Authors:** Omar M. Amin, Richard A. Heckmann, R. Paul Evans, Yahya Tepe

**Affiliations:** 1 Institute of Parasitic Diseases 11445 E. Via Linda # 2-419 Scottsdale Arizona 85259 USA; 2 Department of Biology, Brigham Young University 401 WIDB Provo Utah 84602 USA; 3 Department of Microbiology and Molecular Biology, Brigham Young University 3139 Life Sciences Building Provo Utah 84602 USA; 4 Department of Biology, Faculty of Science, Ataturk University Erzurum 25240 Turkey

**Keywords:** Acanthocephala, *Echinorhynchus baeri*, Neotype, Turkey, *Salmo trutta*, Evolutionary history, DNA analysis, Hook X-ray microanalysis

## Abstract

A population of *Echinorhynchus baeri* Kostylew, 1928 with 18–24 rows of 8–10 proboscis hooks each and long fusiform eggs measuring 95–110 × 18–22 μm collected from *Salmo trutta* (Salmonidae) in a branch of the Murat River in Turkey is described and specimens are designated as neotype. Specimens of two similar populations of *E. baeri* (*E. baeri* Kostylew, 1928 and *E. sevani* Dinnik, 1932) were previously described from *Salmo ischchan* in Lake Sevan, Armenia. Waters of Lake Sevan and the Murat River were previously joined during the Middle Miocene-Pliocene. The two populations from Lake Sevan and ours from Turkey had identical morphology and size eggs. The proboscis armature and eggs, among other features of our Turkish specimens, proved intermediate between *E. baeri* and *E. sevani*, thus eliminating the significance of the described differences between these two species and confirming their synonymy with priority to *Echinorhynchus baeri* (junior synonym: *Echinorhynchus sevani* Dinnik, 1932). *Echinorhynchus baeri* is apparently a highly variable species. The two descriptions from Lake Sevan did not include features or illustrations of females, except for references to trunk and egg size but the eggs were illustrated. Complete morphometric comparisons are made and females of the Turkish material are described for the first time. DNA sequencing (mitochondrial cytochrome oxidase subunit I gene; nuclear *18S rRNA* gene) results from two available *E. baeri* individuals were equivocal. New features to the Acanthocephala include the presence of rootless uncalcified apical proboscis hooks studied with X-ray microanalysis.

## Abstract (fr)

Une population d’*Echinorhynchus baeri* Kostylew, 1928 avec 18–24 rangées de 8–10 crochets au proboscis et des œufs fusiformes allongés mesurant 95–110 × 18–22 μm collectés de *Salmo trutta* (Salmonidae) dans une branche de la rivière Murat en Turquie est décrite et des spécimens sont désignés comme néotypes. Des spécimens de deux populations similaires de *E. baeri* (*E. baeri* Kostylew, 1928 et *E. sevani* Dinnik, 1932) ont été décrits précédemment à partir de *Salmo ischchan* dans le lac Sevan, en Arménie. Les eaux du lac Sevan et de la rivière Murat étaient auparavant jointes au Miocène moyen-Pliocène. Les deux populations, du lac Sevan et les nôtres de Turquie, avaient des œufs de morphologie et de taille identiques. L’armature du proboscis et les œufs, parmi d’autres caractéristiques de nos spécimens turcs, se sont avérés intermédiaires entre *E. baeri* et *E. sevani*, éliminant ainsi la signification des différences décrites entre ces deux espèces et confirmant leur synonymie avec priorité à *Echinorhynchus baeri* (synonyme junior : *Echinorhynchus sevani* Dinnik, 1932). *Echinorhynchus baeri* est apparemment une espèce très variable. Les deux descriptions du lac Sevan ne comprenaient pas de caractéristiques ou d’illustrations de femelles, sauf pour la taille du tronc et des œufs, mais les œufs avaient été illustrés. Des comparaisons morphométriques complètes sont effectuées et des femelles du matériel turc sont décrites pour la première fois. Le résultat du séquençage de l’ADN (gène de la sous-unité I de la cytochrome oxydase mitochondriale, gène nucléaire de l’ARNr *18S*) provenant de deux individus d’E*. baeri* disponibles a été équivoque. Des caractéristiques nouvelles pour les Acanthocephala comprennent la présence de crochets apicaux sans racine au proboscis, non calcifiés, étudiés par microanalyse aux rayons X.

## Introduction

Two populations diagnosed as *Echinorhynchus baeri* Kostylew, 1928 (syn. *Echinorhynchus sevani* Dinnik, 1933, *fide* Platonova, 1963; Bauer, 1987; Amin, 1985, 2013) and *Echinorhynchus sevani* Dinnik, 1932 were collected from the Sevan trout, *Salmo ischchan* Kessler, in Lake Sevan, central eastern Armenia by Kostylew [11] and Dinnik [8]. These two populations of acanthocephalans have occasionally been placed in the genera or subgenera *Metechinorhynchus* Petrochenko, 1956 or *Pseudoechinorhynchus* Petrochenko, 1956 by various authors but they are now recognized as synonyms (see Amin [3]).

**Figure 1.**
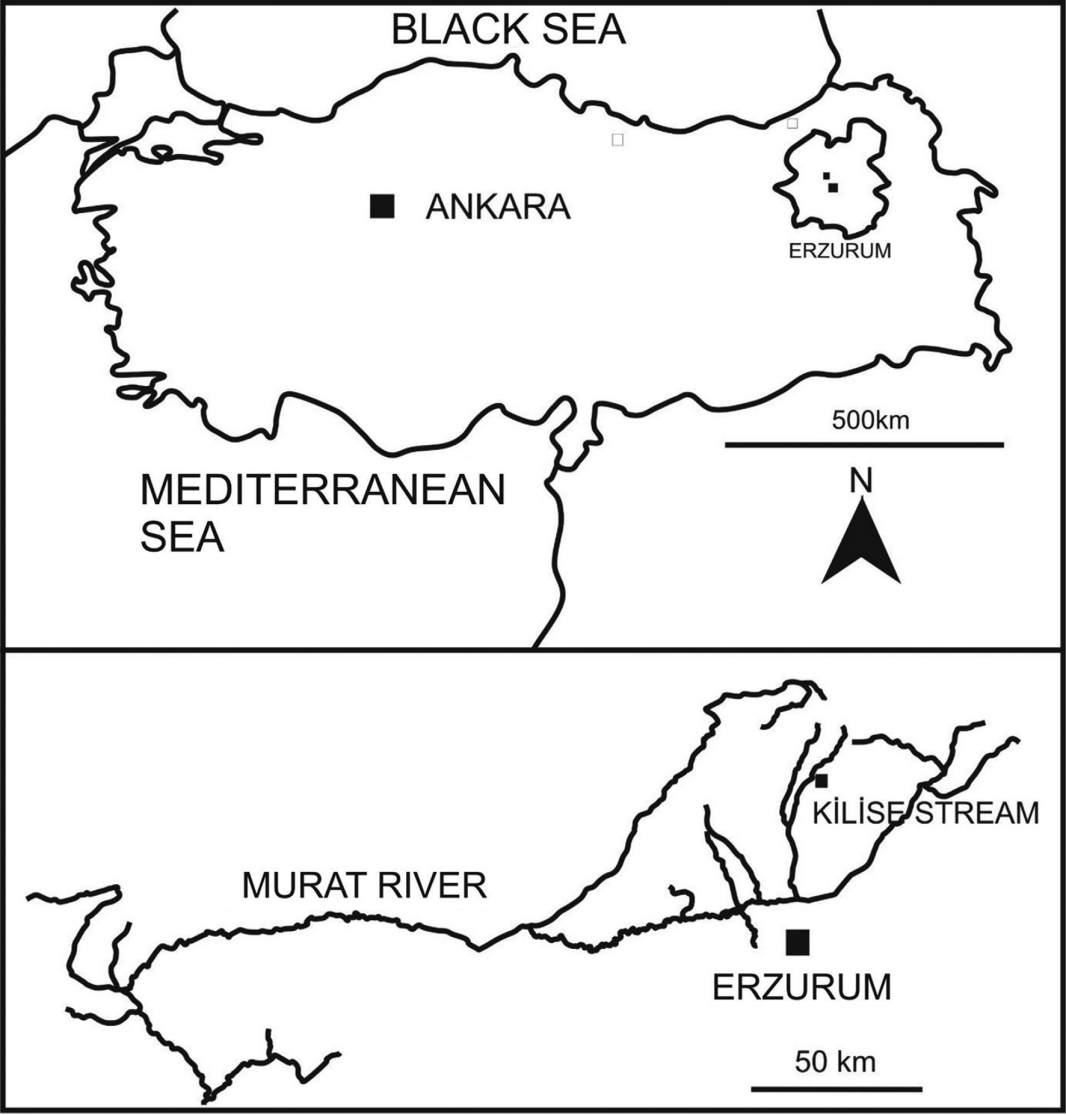
Collection site of *Echinorhynchus baeri* from *Salmo trutta* in the Kilise Stream, Murat River, Turkey.

**Figure 2.**
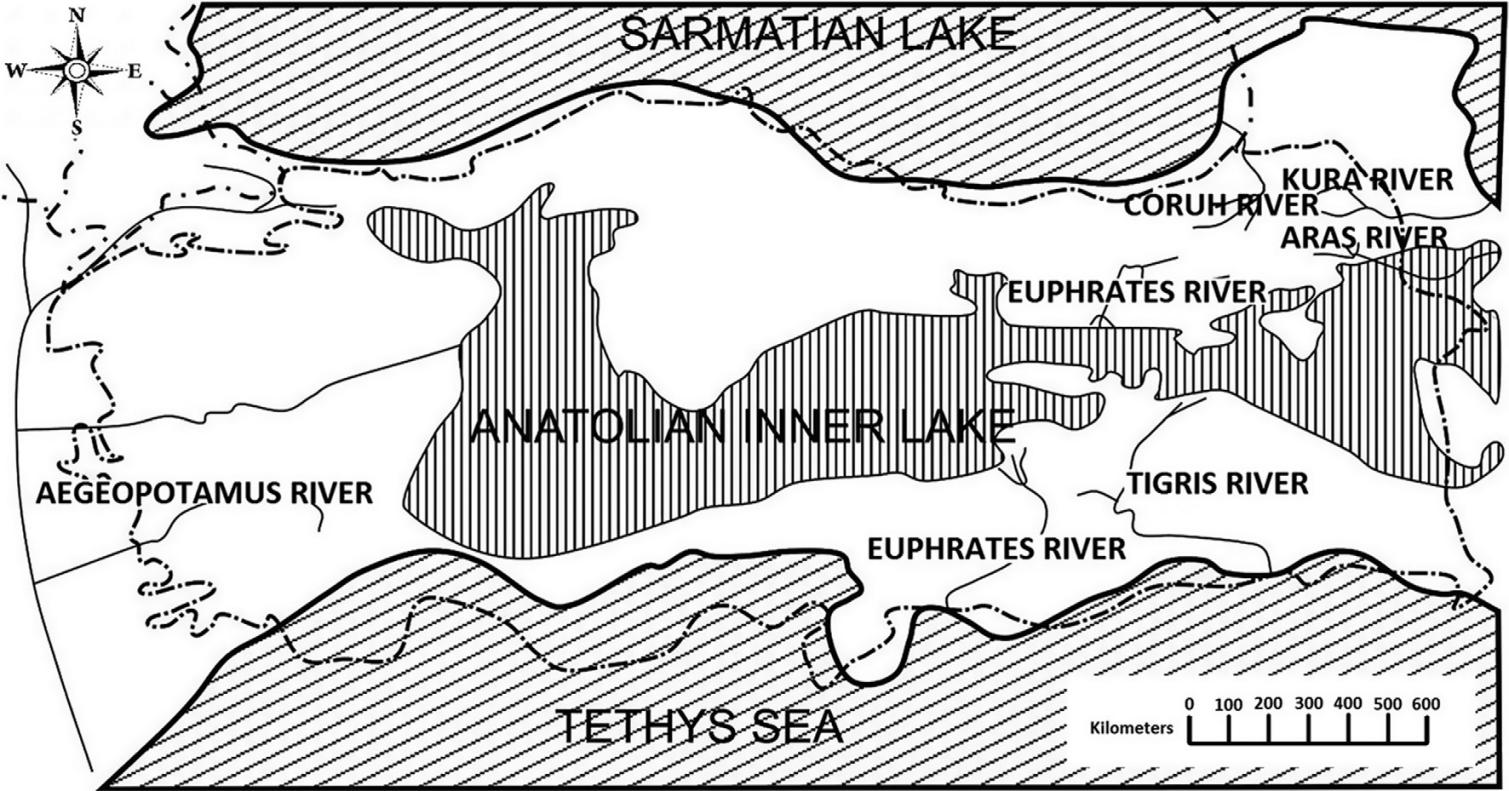
The drainage system of the historic Inner Anatolian freshwater Lake of the Middle Miocene-Pliocene period based on Demirsoy (2008). The drainage is shown to include the Aras, Murat, and Euphrates rivers. Striped lines mark the present borders and coasts of Turkey.

Lake Sevan is the largest lake in Armenia and the Caucasus region and is one of the largest freshwater high-altitude lakes in the world (1,900 m above sea level). It is about 940 km^2^ and its basin covers about 5,000 km^2^. It is fed by 28 rivers and streams but is drained at its northwest reaches by the Hrazdan (Razdan) River which flows south through Yerevan, Armenia’s capital, to join the Aras River in the Ararat plain along the border with Turkey [14]. The Aras River in Turkey rises south of Erzurum in the Bingöl Dağrali mountains [1] near the Kilise Stream of the Murat River where our Turkish specimens of *Echinorhynchus baeri* were collected (Fig. 1) from the brown trout, *Salmo trutta* Linn. Waters of the Aras and the Murat rivers were once connected in earlier geological times (Fig. 2) [7]. The present report discusses the relationship between these three forms, confirms the synonymy of *E. baeri* and *E. sevani* with our new material from Turkey, documents the findings using comparative morphometrics and scanning electron microscopy (SEM), and proposes a scenario of a possible evolutionary relationship among the three forms studied.

## Materials and methods

Brown trout, *S. trutta*, from the Kilise Stream, a branch of the Murat River near Erzurum, Turkey (Fig. 1) (40°5′47.57″ N, 41°11′26.18″ E), were examined for parasites in June 2013 and June, 2015. The intestines of many of these fish were heavily infected with the acanthocephalans reported in this study.

For microscopical examination: freshly collected specimens of various sizes were placed in water overnight or until fully extended then fixed in cold 70% ethanol. Worms were punctured with a fine needle and subsequently stained in Mayer’s acid carmine, destained in 4% hydrochloric acid in 70% ethanol, dehydrated in ascending concentrations of ethanol (24 h each), and cleared in 100% xylene then in 50% Canada balsam and 50% xylene (24 h each). Whole worms were then mounted in Canada balsam. Measurements are in micrometers, unless otherwise noted; the range is followed by the mean values between parentheses. Width measurements represent maximum width. Trunk length does not include proboscis, neck, or bursa. Line drawings were created using a Ken-A-Vision microprojector (Ward’s Biological Supply Co., Rochester, NY, USA) which uses cool quartz iodine 150 W illumination. Color-coded objectives, and 10X, 20X, and 43X lenses, are used. Images of stained whole mounted specimens are projected vertically on 300 series Bristol draft paper (Strathmore, Westfield, MA, USA), then traced and inked with India ink. Projected images are identical to the actual specimens being projected. The completed line drawings are subsequently scanned at 600 pixels on a USB and subsequently downloaded on a computer.

For SEM studies: specimens previously fixed in 70% ethanol were placed in critical-point drying baskets and dehydrated using ethanol series of 95% and 100% for at least 10 min per soak followed by critical-point drying [12]. Samples were mounted on SEM sample mounts, gold coated, and observed with a scanning electron microscope (FEI Helios Dual Beam Scanning Electron Microscope, Hillsboro, OR, USA). Digital images of the structures were obtained using digital imaging software attached to a computer.

For X-ray microanalysis (XEDS), standard methods for SEM preparation [12] were used. Coated specimens were examined with an FEI Helios Dual Beam Scanning Electron Microscope equipped with an Apollo 40 Silicon Drift Detector (SDD) X-ray detector (FEI, Hillsboro, OR, USA). X-ray spot analysis and line scan analysis were performed at 15 kV and results were represented in charts and recorded on digital imaging software attached to a computer. Results were recorded as weight percent and atom percent for the chemical elements. The cutting of each hook was accomplished with a gallium beam using the FEI Helios Dual Beam Electron Microscope. Both a normal hook and the very small hook at the tip of the proboscis were cut and then analyzed for chemical elements. The hook was positioned at the eccentric position of the stage and cut longitudinally using a 30 kV gallium ion gun operating at 2.8 nA. A cross-sectional pattern was used followed by a cleaning cross-section to provide a clean cut surface to image. Images were of the cut surface using a 5 kV electron beam at 0.17 nA followed by X-ray spectrum analysis using a 15 kV electron beam. The Energy Disruptive X-Ray Analysis (EDXA) Genesis system was performed utilizing the Apollo 40 SDD (Silicon Drift Detector) X-ray detector (FEI, Hillsboro, OR, USA) with results stored with a USB.

DNA was separately extracted from two ethanol preserved (70%) specimens using a Qiagen DNAeasy Blood and Tissue Kit (Qiagen Inc., Valencia, CA, USA). Entire individuals were soaked in 500 μL of ATL buffer for 10 min prior to DNA digestion. Samples were macerated by scissors and the protocol was followed, as outlined by the manufacturer.

A 664-bp fragment of the mitochondrial cytochrome oxidase subunit 1 gene (CO1) was PCR amplified using the primers 5′-AGTTCTAATCATAA(R)GATAT(Y)GG-3′ and 5′-TAAACTTCAGGGTGACCAAAAAATCA-3′ [9]. Primers used for the amplification of a 1685-bp fragment of the nuclear *18S* ribosomal RNA gene (*18S*) were 5′-AGATTAAGCCATGCATGCGTAAG-3′ and 5′-TGATCCTTCTGCAGGTTCACCTAC-3′ [15]. Reaction cocktails were 12.5 μL in volume and included the following reagents: DNA template (~150 ng), nuclease free water (2.25 μL), oligonucleotide primers (10 pmol each), and Promega GoTaq^®^ Green Master Mix (6.25 μL). The thermal profile began with an initial denaturation step of 95 °C for 2 min to activate the enzyme, followed by 35 cycles at 95 °C for 30 s, 55 °C for 30 s, and 72 °C for 90 s, and concluded by a rapid cool down to 4 °C. Successful amplifications were verified qualitatively by viewing PCR products under ultraviolet radiation following electrophoresis through 1.0% agarose gels. Millipore MultiScreen_μ96_ filter plates were used to purify PCR products, following the manufacturer’s recommended protocol.

Cycle sequencing reactions were performed using the ABI BigDye Terminator Protocol (Applied Biosystems, Foster City, CA, USA). Reaction cocktails were 10.5 μL in volume and were mixed using the following reagent amounts: purified PCR product (~150 ng), nuclease free water (2.75 μL), 5 × Tris buffer (1.75 μL), primer (6 pmol), and dye terminator reaction mix (0.5 μL). Both DNA strands were sequenced using the same primers that were used to amplify the genes via PCR. The thermal profile for the sequencing reactions consisted of 25 cycles at 96 °C for 10 s, 50 °C for 5 s, 60 °C for 4 min, followed by a rapid cool down to 4 °C. All sequencing was carried out on an ABI 3730xl automated sequencer in the DNA Sequencing Center at Brigham Young University.

All samples showed greater than 50% double peaks on the sequence electropherograms for both the CO1 and *18S rRNA* genes. The original PCR products and a second independent amplification using the same procedure as described above were individually cloned using a TOPO TA-Cloning kit (Invitrogen). PCR products were ligated into vectors (pCR 2.1-TOPO) and used to transform chemically competent *Escherichia coli* cells by heat shock at 42 °C. After growth in S.O.C. medium (Invitrogen) at 37 °C for 1 h, transformed cells were selected by plating on LB medium supplemented with 50 μg/mL Ampicillin and 50 μL of X-gal (40 mg/mL). White colonies (transformed cells) were picked and diluted in 100 μL water. DNA was extracted by heating to 100 °C for 3 min. DNA inserts were sequenced using the M13 primers included in the kit.

Attempts to locate Armenian specimens of *E. baeri* collected and reported by Kostylew [11] and Dinnik [8] in Georgia, Russia, Armenia, and Germany for molecular comparisons were unsuccessful. Additionally, no specimens were found in the collections of the Schmalhausen Institute of Zoology, National Academy of Sciences of Ukraine, the Institute of Zoology of the National Academy of Sciences of Armenia at Yerevan, Yerevan State University, the Division of Natural Sciences, Scientific Center of Zoology, the Hydroecology Institute of Zoology, National Academy of Sciences, Yerevan, the Museum für Naturkunde Leibniz-Institut für Evolutions- und Biodiversitätsforschung, Berlin, Germany, the Senckenberg Research Institute and Natural History Museum Frankfurt, Germany, and the Anschrift Biozentrum Grindel und Zoologisches Museum, University of Hamburg, Germany.

No references to deposited material were made in the Kostylew [11] or the Dinnik [8] reports.

## Results

Eighty-four individuals of *S. trutta* from the Kilise stream, a branch of the Murat River, near Erzurum, Turkey, were examined for parasites. A total of 623 specimens of *E. baeri* were collected from 71 fish with a mean of 8.77, a median of 5.00, and a mean abundance of 7.42. The variance/mean ratio was 19.65, suggesting an overdispersed distribution. These Turkish specimens are described below.

## *Echinorhynchus baeri* Kostylew, 1928

Family Echinorhynchidae Yamaguti, 1935

Genus *Echinorhynchus* Zoega in Müller, 1776

Host: Brown trout, *Salmo trutta* Linn. (Salmonidae).

Other host: Sevan trout, *Salmo ischchan* Kessler (Salmonidae) [8, 9].

Site in host: Intestine.

Specimens: Six slides of whole-mounted male and female specimens were deposited in the parasite collection of the Harold W. Manter Laboratory (HWML) collection no. 101,847 at the University of Nebraska State Museum, Lincoln, NE, USA.

Locality: Kilise Stream, Murat River near Erzurum, Turkey (40°5′47.57″ N, 41°11′26.18″ E).

Other locality: Lake Sevan, Armenia [8, 9].

Comments: Considering the absence/loss of any type material of this species, the present material from Turkey is designated as neotype.

### Description (Figs. 3–22)

**Figures 3–10.**
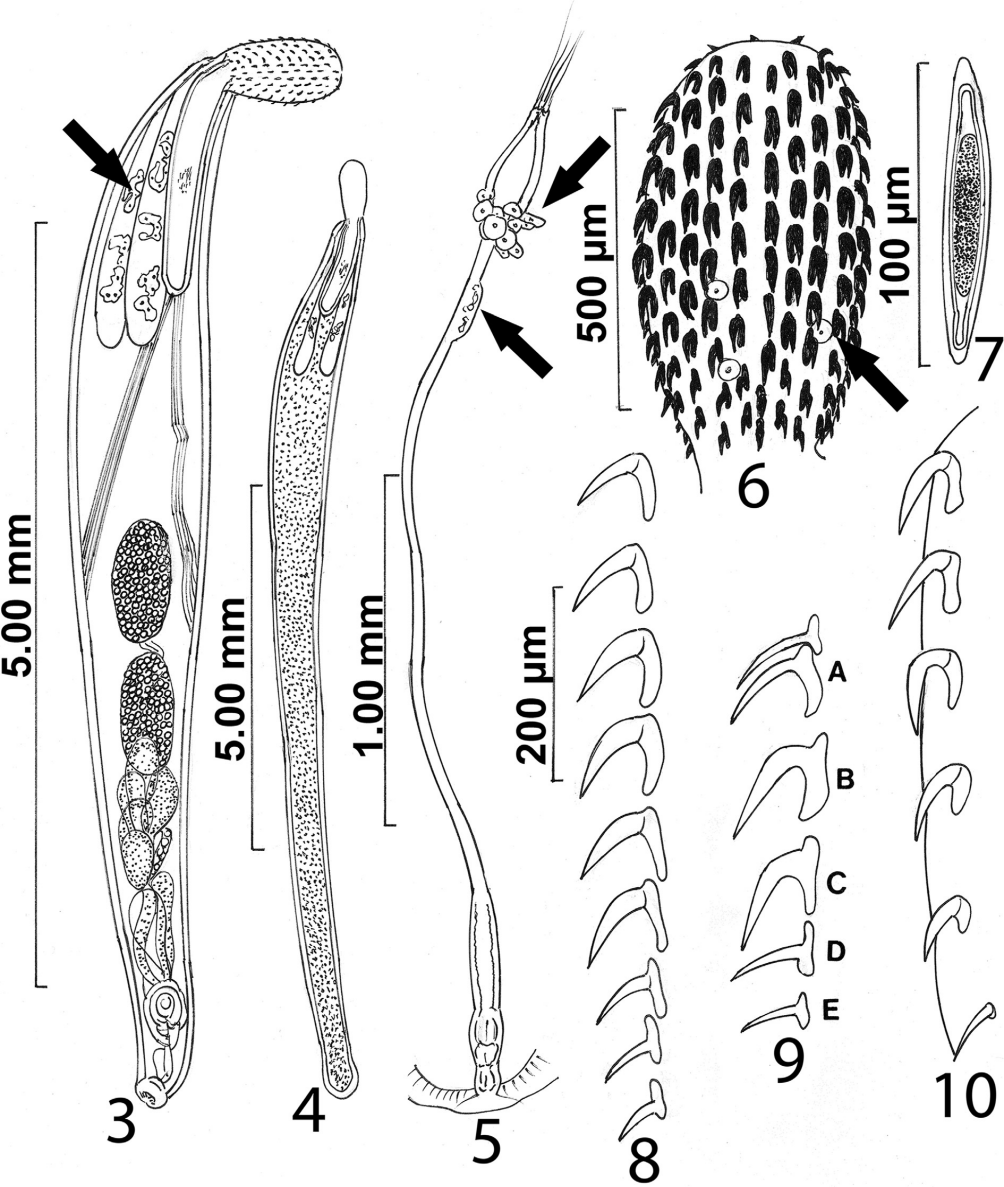
Specimens of *Echinorhynchus baeri* collected from *Salmo trutta* in Turkey and proboscis hook rows of specimens of *E. sevani* and *E. baeri,* respectively*,* collected from *Salmo ischchan* in Lake Sevan, Armenia. **3.** A male specimen. Note the unique amoeboid, lobulated giant nuclei in the long lemnisci (arrow), the prominent retractor muscles, and the near contiguous ovoid-elongate testes. Proboscis is usually bent ventrad. **4.** A gravid female with typically long lemnisci. The reproductive system is obscured by eggs. **5.** The female reproductive system. Note the very long and slender uterus and the longitudinal bulge near its distal end (upper arrow). Also note the laterally extending uterine glands at the base of the uterine bell (lower arrow). **6.** The proboscis of the male specimens in Fig. 3. Note the uninucleated round cells (arrow). **7.** A ripe egg with prominent polar prolongation of the fertilization membrane. **8.** A ventral row of proboscis hooks from a male specimen. Note the lack of root manubria anteriorly and the gradual development of manubria with decreasing size of roots posteriorly. **9.** Lateral view of hooks of *E. sevani* after Dinnik (1932) showing variable manubriation in all hook roots “A = first two hooks. B & C = middle hooks, D & E = last two hooks of the vertical row.” Measurement bars were not provided. **10.** Lateral view of hooks of *E. baeri* after Kostylew (1928) showing the absence of manubria in all hook roots and the virtual absence of roots of the basal hook; measurement bars were not provided.

**Figures 11–16:**
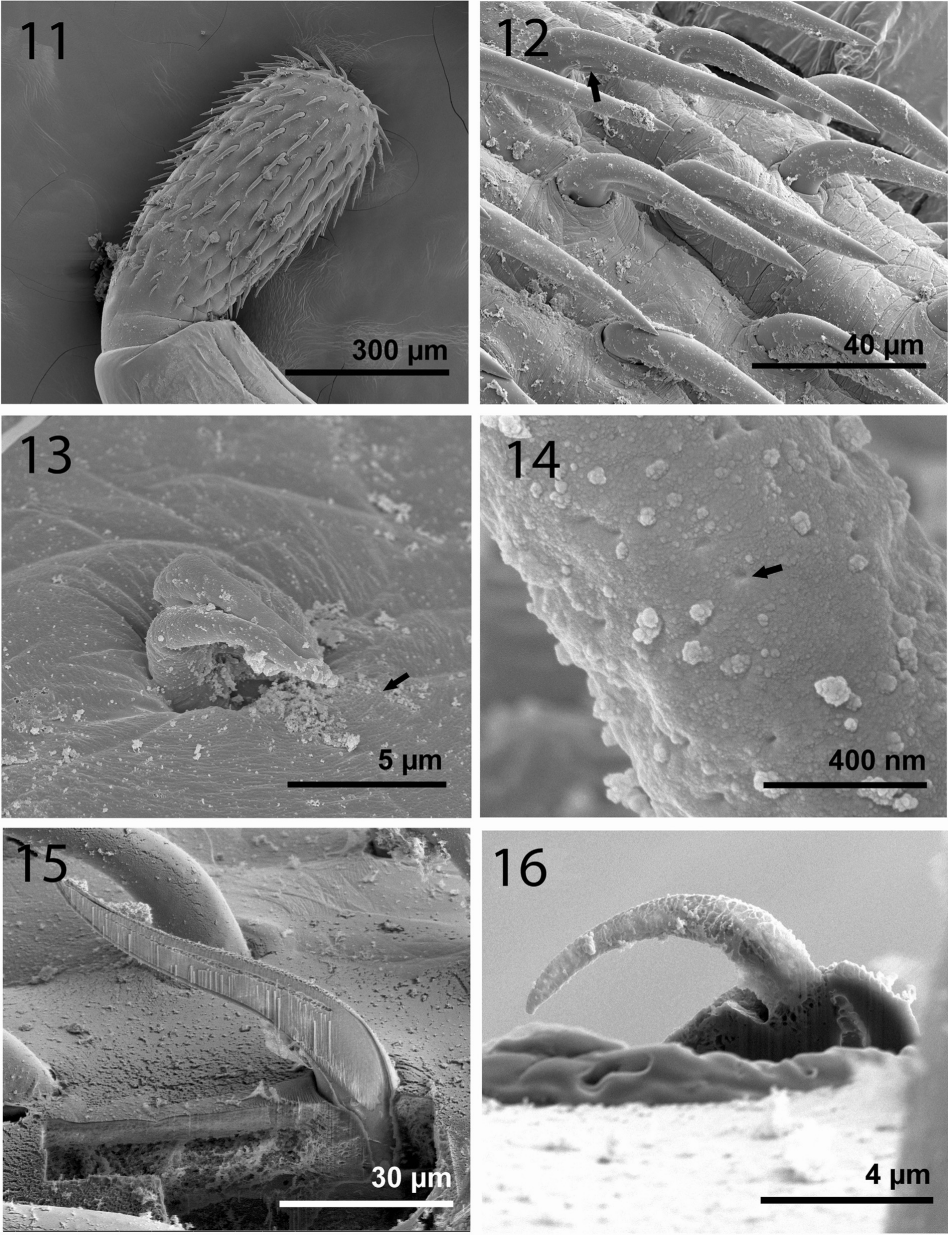
SEM of mature specimens of *Echinorhynchus baeri* from *S. trutta* in Turkey. **11.** Proboscis of a female specimen. Note variation in hook size; smaller hooks at base **12.** Anterior hooks. Note indentation at the base of the hooks (arrow). **13.** Double miniature hooks at apical end of proboscis (arrow); occasionally one miniature apical hook present. **14.** Higher magnification of an apical hook; note perforations. This hook has a low Ca reading (see EDAX data). **15.** A gallium cut normal hook from the mid-proboscis. Note prominent calcified root. **16.** A gallium cut miniature apical hook. Note the hollow base and absence of roots.

**Figures 17–22.**
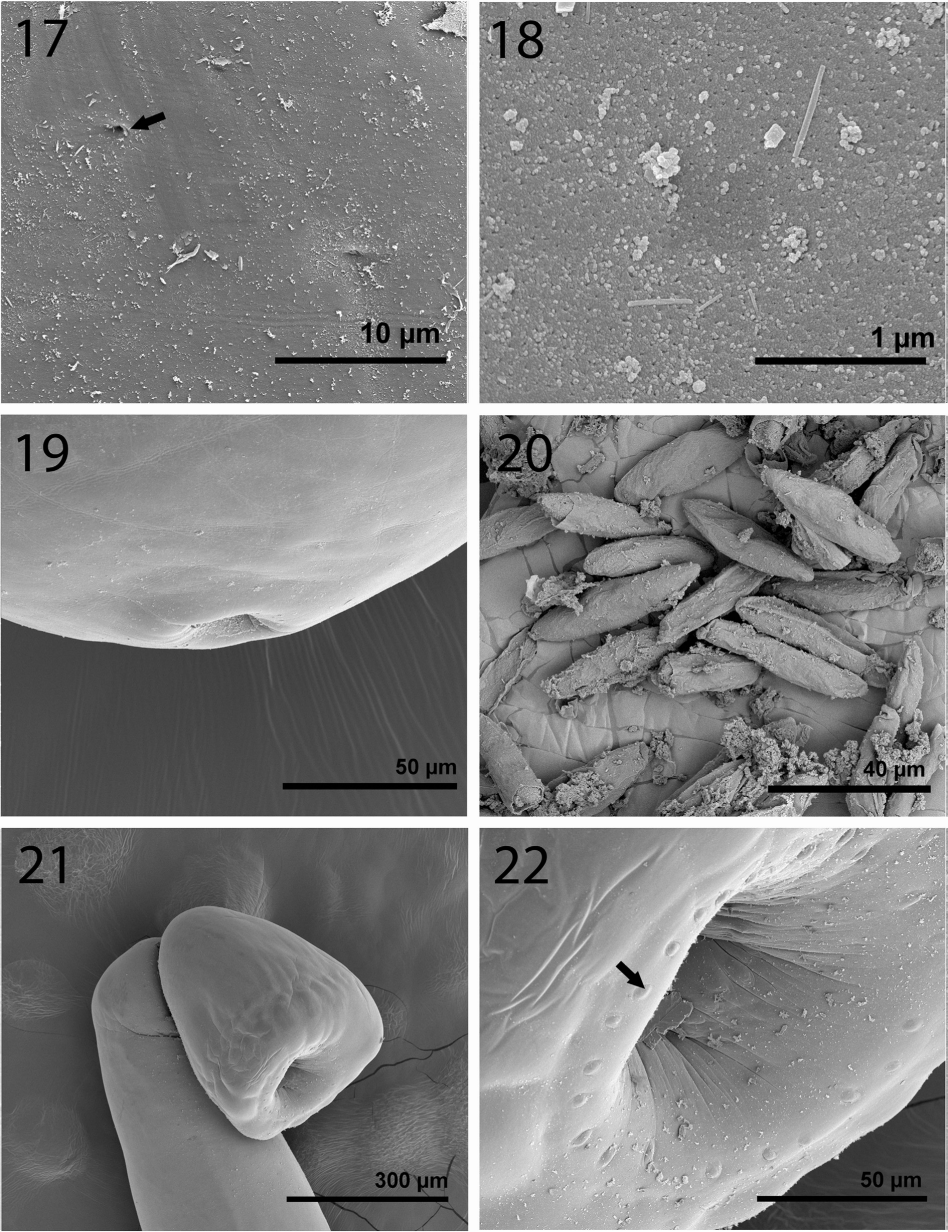
SEM of mature specimens of *Echinorhynchus baeri* from *S. trutta* in Turkey. **17.** Sensory pore (arrow) at the base of the proboscis. No micropores here. **18.** Epidermal micropores at midtrunk. **19.** The posterior end of a female specimen showing terminal gonopore. **20.** Egg mass from a dissected female specimen. **21.** Bursa of a male specimen. **22.** The opening of the bursa showing one ring of sensory knobs (arrow).

**Figure 23.**
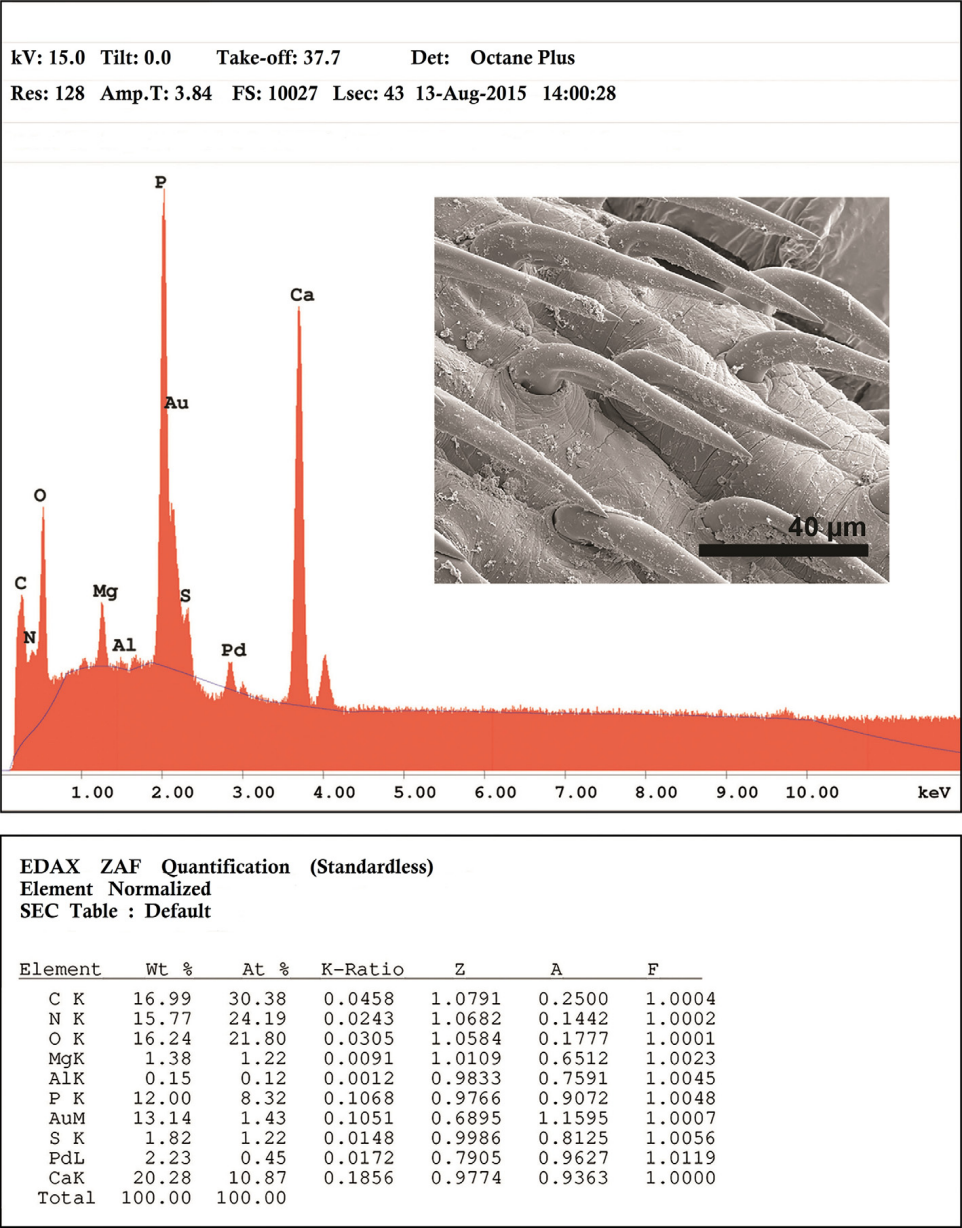
The printout of the elemental scan (EDXA) for the common large hooks for *E. baeri*. Note height of calcium and phosphorus peaks.

**Figure 24.**
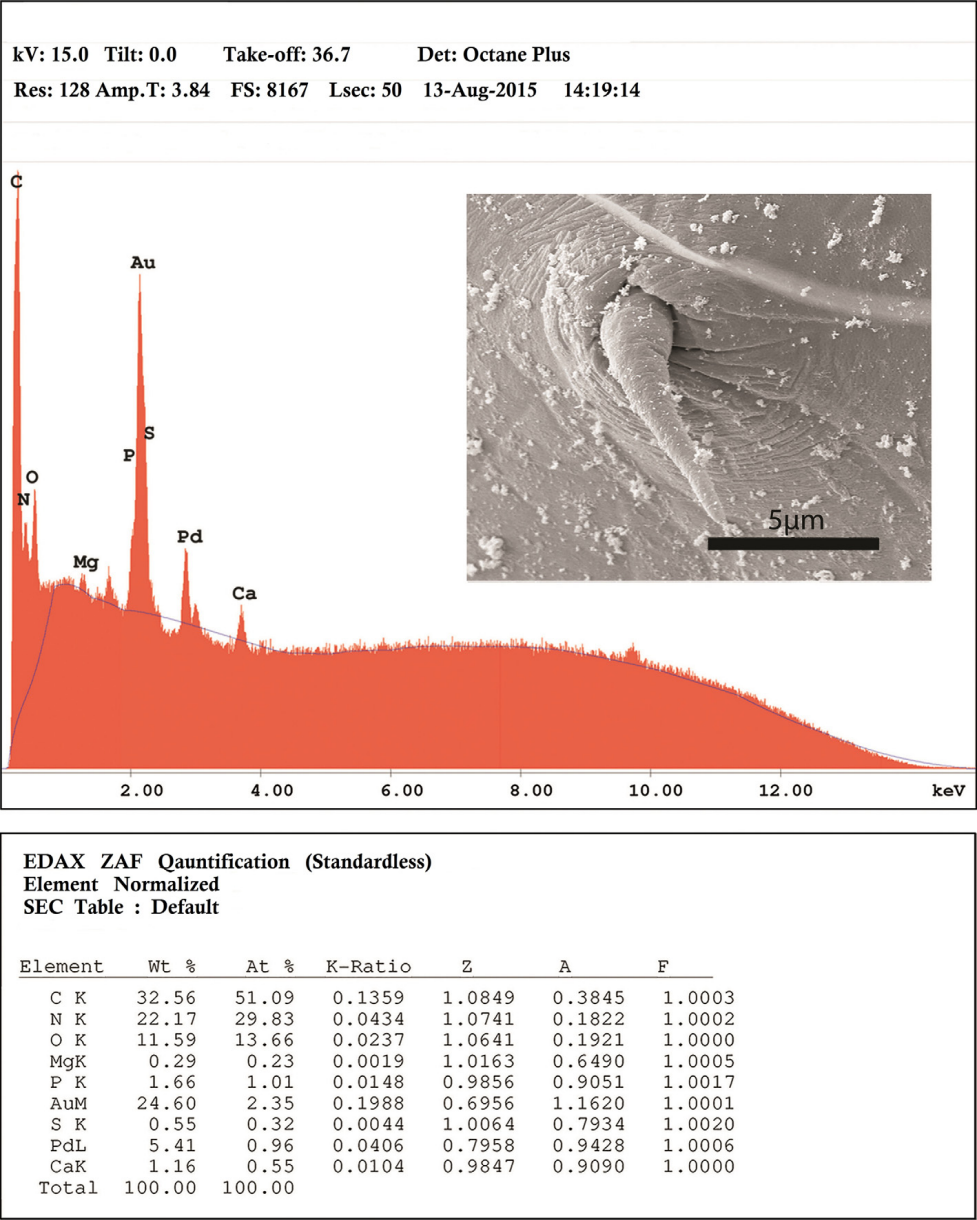
The printout for the elemental scan (EDXA) for the miniature apical hook at the apex of the *E. baeri* proboscis. Note the drop in calcium and phosphorus peaks compared to that of normal hooks (Fig. 23).

*General.* With characters of the genus *Echinorhynchus*. Shared structures invariably larger in females than in males. Trunk cylindrical, widest in anterior third, and gradually tapering posteriorly; females with slightly expanded rounded posterior end (Figs. 3, 4). Body wall with numerous multinucleated amoeboid to round elongate cells, oriented laterally, and micropores with diverse diameter and distribution in all trunk regions (Fig. 18) including the female genital orifice and the bursa. Base of proboscis with sensory pores but no micropores (Fig. 17). Proboscis cylindrical, plump, rounded anteriorly, and widest at middle (Fig. 11), often tilted ventrad (Fig. 3), with three or more large uninucleated round cells mostly in posterior half (Fig. 6, arrow) and apical rootless uncalcified hooks with multiple perforations (Figs. 13, 14, 24). Proboscis with 18–24 rows with 8–10 alternating hooks each (rarely 11 in 1 male) with normal levels of structural minerals (Fig. 23). Occasionally, whole range of 8–10 hooks per row on individual proboscides. Hooks more robust and slightly longer ventrally than dorsally and transition from small anteriorly to largest at middle (hooks 3–6 from anterior) then smallest basally. Anterior hooks with indentations near base (Fig. 12, arrow). Anterior and middle hooks with simple roots, about as long as blades, directed posteriorly. Posterior hook roots (nos. 6–10 from anterior) with manubria varying from small (no. 6) to prominent (no. 10) with gradually decreasing size of roots posteriorly (Fig. 8). Neck marked. Proboscis receptacle double-walled with cephalic ganglion at middle and with two sets of prominent retractor muscles attached to midtrunk (Fig. 3). Lemnisci usually subequal, digitiform, invariably and markedly longer than receptacle, widening posteriorly, with at least three large, multinucleated, lobulated giant nuclei each (arrow), and with posterior fibrous connective. Gonopores terminal in both sexes.

*Male* (based on 21 whole mounted mature adults with sperm, and 5 specimens studied by SEM). Measurements and counts in Tables 1 and 2. Testes ovoid-elongate, almost equal, close or contiguous, equatorial or slightly postequatorial. Cement glands clustered to paired, contiguous with posterior testis or occasionally overlapping it (Fig. 3). Anterior cement glands larger than posterior glands emptying into cement ducts in two groups surrounding common sperm duct and joining posteriorly. Saefftigen’s pouch prominent, overlapping cement ducts (Fig. 4). Bursa muscular, thick walled, directed ventrad, with one ring of sensory structures (Figs. 21, 22).

**Table 1.** A comparison between the three populations of *Echinorhynchus baeri* from Lake Sevan and Turkey.

	Lake Sevan	Lake Sevan	Turkey
	Kostylew, 1928	Dinnik, 1933	this paper
Host	*Salmo ischchan*	*Salmo ischchan*	*Salmo, trutta*
Location	Lake Sevan, Armenia	Lake Sevan, Armenia	Kilise Stream, Murat River, Turkey
Males			
Trunk mm	7.25 × 0.60–0.75	3.5–5.5 × 0.5–0.7	5.62–7.37 (6.44) × 0.62–0.87 (0.75)
Proboscis μm	770 × 300–380	600–1,000 × 300–500	686–728 (706) × 281–364 (329)
Hooks^a^	All slender	Median hooks more robust	Slender with occasional robust hooks
	10 hooks in 22–24 rows	8–9 hooks in 18–20 rows	8–10 (rarely 11) (9.0) hooks in 18–24 (21.9) rows
Median hooks^a^	Dorso-ventrally differentiated	Not differentiated	Some dorso-ventral differentiation
Hook roots^a^	Simple, posterior hook rootless	Manubriated	Simple anteriorly, manubriated posteriorly
Receptacle mm	1.40	1.10–1.50 × 0.20–0.40	1.09–1.38 (1.23) × 0.25–0.44 (0.34)
Lemnisci mm^a^	Not longer than receptacle	Shorter than receptacle, 0.67–1.0	Sub equal; markedly longer than receptacle
			Shorter lemniscus 1.35–1.77 (1.53) × 0.13–0.40 (0.19)
			Longer lemniscus 1.46–1.89 (1.64) × 0.15–0.31 (0.21)
Testes	Round, postequatorial,	Elongate, equatorial,	Elongate, equatorial to postequatorial,
	Not contiguous	Contiguous	Nearly contiguous
Ant. testis μm	550–700 (?) × 300–380	350–420 × –b	541–853 (713) × 260–416 (291)
Post. testis μm	380–500 × 300–320	350–420 × –	541–1,040 (723) × 270–395 (315)
Cement glands	6 in pairs	6 clustered	In various patterns between paired & clustered
Dimensions μm	–	–	Anterior: 312–572 (461) × 208–406 (283)
			Posterior: 312–520 (388) × 218–291 (245)
Saefftigen’s pouch μm	–	–	520–624 (559) × 187–302 (250)
Females			
Trunk mm	11.00–12.00 × 0.60–0.75	6.50–14.00 × 0.70–1.00	8.17–14.50 (11.86) × 0.70–1.20 (0.89)
Proboscis μm	–	–	728–894 (820) × 343–458 (395)
Hooks	–	–	8–10 (8.6) hooks in 18–24 (21.6) rows
Receptacle mm	–	–	1.30–1.82 (1.52) × 0.21–0.44 (0.36)
Lemnisci mm	–	–	Shorter: 1.25–2.45 (2.01 × 0.16–0.29 (0.23)
			Longer: 1.51–2.50 (2.12) × 0.16–0.29 (0.22)
Reproductive system mm	–	–	1.98–3.56 (2.76), 20–29 (24%) of trunk length
Eggs μm	105–126 × 22–24	108–120 × 19–22	95–110 (105) × 18–22 (20)

a Descriptive characters apply to males and females.

b Not given.

**Table 2.** Measurements of the proboscis hook blades of *Echinorhynchus baeri* from Lake Sevan and from Turkey.^a^

	Males						Females					
Hook	Dorsal			Ventral			Dorsal			Ventral		
No.	LSK^b^	LSD	Turkey	LSK	LSD	Turkey	LSK	LSD	Turkey	LSK	LSD	Turkey
1.	–	83	77–85 (81)	–	83	70–87 (78)	–	78	72–97 (86)	–	78	85–92 (75)
2.	–	91	82–92 (86)	–	91	82–87 (84)	–	91	87–100 (95)	–	91	88–102 (96)
3.	73	99	87–92 (90)	78	99	85–92 (88)	73	93	87–102 (95)	78	93	90–110 (102)
4.	73	104	80–92 (87)	82	104	85–92 (88)	73	99	95–105 (99)	82	99	92–112 (100
5.	92	108	82–97 (88)	87	108	85–92 (86)	92	104	95–107 (99)	87	104	92–110 (100)
6.	78	99	70–87 (82)	92	99	82–87 (85)	78	83	95–100 (96)	92	83	97–110 (101)
7.	64	78	62–82 (70)	92	78	75–92 (80)	64	74	80–90 (85)	92	74	82–105 (93)
8.	46	66	60–70 (65)	64	66	60–72 (67)	46	66	67–72 (70)	64	66	67–92 (82)
9.	46	50	45–60 (54)	46	50	52–57 (55)	46	62	52–72 (64)	46	62	64–87 (73)
10	46	–	42–55 (48)	46	–	–	46	–	–	46	–	62–67 (65)

a In *E. baeri* from Lake Sevan (Kostylew, 1928), hook measurements were not separated by sex. In *E. baeri* from Lake Sevan (Dinnik 1932), they were not separated by dorsal vs. ventral.

b LSK: *Echinorhynchus baeri* (Kostylew, 1928); LSD: *E. baeri* (Dinnik, 1932).

*Female* (based on 26 whole mounted mostly gravid adults, and 5 specimens studied by SEM). Measurements and counts in Tables 1 and 2. Reproductive system about ¼ trunk length. Uterus unusually long and slender compared to rest of the reproductive system (Fig. 5); its length proportional to trunk length. Vagina without prominent sphincters. Proximal end of uterine bell with few laterally projecting nucleated cells (Fig. 5, top arrow) and basal expansion (Fig. 5, bottom arrow). Gonopore terminal with plain non-specialized orifice (Fig. 19). Eggs elliptoid elongate, non-ornate, with marked polar prolongation of fertilization membrane (Figs. 7, 20).

### Molecular analysis

For DNA sequence analysis, only two individuals were available for DNA extraction. DNA extraction was routine, yielding ~20 micrograms of high-quality DNA per individual with spectrophotometric 260 nm/280 nm absorbance ratios greater than 1.8 and a preponderance of high molecular weight DNA as determined by agarose gel electrophoresis. The DNA sequencing electropherograms of *COX1* and *18S* genes from three separate PCRs from both individuals showed greater than 50% double peaks. To resolve the apparent admixture of DNA sources, PCR products were individually cloned using TA cloning and a minimum of 10 clones each were sequenced for the *COX1* and *18S rRNA* genes from each of the two individuals.

### X-ray microanalysis of hooks

An X-ray elemental analysis of the normal common and the miniature apical hooks is compared (Table 3, Figs. 13, 14, 23, 24). The amount of calcium (Ca), phosphorus (P), and sulfur (Table 3) is emphasized because they metabolize into hardened structures as found in mammalian teeth. Other elements not included in Table 3 are assumed since they are present in all protoplasm (Figs. 23, 24).The Au and Pd are coating materials mentioned in the Materials and Methods section. The apical hooks lack roots and their levels of structural minerals especially calcium, phosphorus, and sulfur are very low (Fig. 24) compared to those in the normal large hooks (Fig. 23).

**Table 3.** Comparison of atomic % and weight % of elements of selected large hooks vs. apical hooks.

	Atomic %		Weight %	
	Common hook	Apical hook	Common hook	Apical hook
Overall for cut hooks				
Calcium (Ca)	10.87	0.34	20.28	0.74
Phosphorus (P)	8.32	0.93	12.00	1.24
Sulfur (S)	1.22	0.61	1.82	1.06
Magnesium (Mg)	1.22	0.21	1.38	0.30
Base of cut hooks				
Calcium (Ca)	1.87	0.55	3.56	1.16
Phosphorus (P)	1.51	1.01	2.21	1.66
Sulfur (S)	1.10	0.32	2.44	0.55
Magnesium (Mg)	0.42	0.23	0.48	0.29

## Discussion

### Origins

The two populations of *E. baeri* from Lake Sevan, Armenia and the one from the Kilise stream, Murat River, Turkey are clearly conspecific. The two Armenian taxa from Lake Sevan, *E. baeri* and *E. sevani*, have already been synonymized [2, 3, 5, 16]. Differences between specimens of these two “species” are further rendered inconsequential with the discovery of the Turkish population that revealed intermediate character states justifying the synonymy. Additional unique features characterize the Turkish specimens. This scenario suggests a common ancestral stock that would have diversified into the Turkish and the Armenian material. Lake Sevan where the Kostylew [11] and Dinnik [8] specimens were collected from *S. ischchan* drains at its northwest reaches by the Hrazdan (Razdan) River which flows south to join the Aras River in the Ararat plain along the border with Turkey [14]. The Aras River in Turkey rises south of Erzurum in the Bingöl Dağrali mountains [1] near the Kilise stream of the Murat River where our Turkish specimens of *E. baeri* were collected (Fig. 1) from *Salmo trutta*. Waters of the Murat River, which feed into the Euphrates, and of the Aras River were once connected via the freshwater Inner Anatolian Lake (IAL) in earlier geological times (Fig. 2). IAL filled the middle of Anatolia (Asia Minor) 10.3 million to 2 million years ago during the Middle Miocene-Pliocene. During this period, the drainage system of IAL included the Aras and Kura rivers that flowed east to the Caspian Sea, and the northern Murat-Euphrates-Tigris rivers that flowed southwards to the Persian Gulf (Fig. 2) [7]. Accordingly, the process of diversification of the present three populations of *E. baeri* from a presumed common IAL-based ancestor would have taken place about 2 million years ago once geographical isolation between the Armenian and Turkish stocks had taken place. Specimens from the two collections from Lake Sevan are different enough to suggest two populations that may have diversified more recently. It is exciting to put a time frame to measure variations in this species as depicted in this study.

### Character types

Three types of characters are identified. The first two character types (similar and intermediate characters) are comparative in nature and can be used for comparing the three populations of *E. baeri.* The third type is represented by novel characters that are unique to the Turkish material and have no comparable states in the two Lake Sevan populations.

#### Similar characters

Characters that were found to be similar in all three sets of specimens (populations) include general morphology and egg size, as well as the shape and size of proboscis, receptacle, and trunk. The trunk of the Dinnik’s (1932) specimens and eggs of the Turkish material were somewhat smaller (Table 1).

#### Intermediate characters

Characters of the Turkish materials that were found to be intermediate between those of the two Lake Sevan populations include proboscis hooks and roots, testes shape and position, and cement gland pattern. The proboscis of our Turkish specimens had 18–24 rows of 8–10 hooks each compared to 22–24 × 10 and 18–20 × 8–9 in the Lake Sevan specimens. Ventral hooks were slightly longer and more robust than dorsal hooks in our Turkish specimens and those of Kostylew [11]. Hooks were longer in females than in males in Turkish specimens but not so in those of Dinnik [8] which exhibited robust median hooks (Table 2). All hook roots of Dinnik’s [8] specimens had manubria to different degrees (Fig. 9). But those of Kostylew [11] were invariably simple and directed posteriorly except for the basal rootless hook (Fig. 10). Roots of the anterior hooks in our Turkish specimens were simple and similar to those of Kostylew’s [11] specimens but those of the posterior hooks had manubria, like those of Dinnik’s [8] specimens (Figs. 8–10). Unlike the round postequatorial non-contiguous testes of Kostylew’s [11] male specimens (Fig. 4 of Kostylew [11]), the testes in the Turkish specimens are invariably elongate and often contiguous like the equatorial testes of the males described by Dinnik [8] (Fig. 13 of Dinnik [8]) but their position varied between equatorial to somewhat postequatorial (Fig. 3). The cement glands in the Kostylew [11] specimens were in longitudinal pairs (Fig. 4 of Kostylew [11]) but were in a cluster in Dinnik’s [8] specimens (Fig. 13 of Dinnik [8]). In our specimens, cement glands were intermediate between the two patterns, never in longitudinal pairs (Fig. 3).

#### Novel characters

New variations were observed in the size and nuclei of the lemnisci and body wall nuclei. The lemnisci were reported to be “not longer than” (about as long as receptacle; Fig. 4 of Kostylew [11]) or “shorter than” the proboscis receptacle (definitely markedly shorter; Fig. 13 of Dinnik [8]) in the Lake Sevan populations. In our Turkish specimens, the lemnisci were markedly to considerably longer than the proboscis receptacle (Figs. 3, 4). In a few cases, they were almost twice as long as the receptacle. The lemnisci in our specimens had three large, amoeboid, lobulated giant nuclei each. This is a unique trait never reported in any echinorhynchid acanthocephalan including the Lake Sevan material, to the best of our knowledge. Giant nuclei of various forms, unlike the ones reported here, are known in the lemnisci and body wall of eoacanthocephalans only. The body wall of our Turkish specimens had numerous multinucleated amoeboid to round elongate cells, oriented laterally and the proboscis has large uninucleated round cells. These latter two traits are unique to the Turkish specimens.

### Other novel characters and X-ray microanalysis of hooks

In a similar EDXA study of the proboscis hooks of another acanthocephalan, *Rhadinorhynchus ornatus Van Cleave*, 1918, Heckmann et al. [10] demonstrated that the base of the hook is flexible with high sulfur content at the tip and sides while the center of the hook was high in calcium and phosphorus. The calcium and phosphorus form a rigid phosphate apatite similar to the enamel of mammalian teeth with disulfide bonds (cysteine) enhancing the strength of the structure. The enamel of mammalian teeth is over 95% inorganic matter representing the hardest tissue in the body [10]. The apical hooks lack roots and their levels of structural minerals especially calcium and phosphorus are too low to have any structural/attachment utility. No such structures have ever been reported in any species of Acanthocephala that we know of. Some of the above unique characters may be novel because they were simply not seen or reported by earlier observers, if they were present in the Lake Sevan material in the first place.

Other new features of the Turkish material include the ring of sensory knobs on the inner orifice of the bursa and the presence of micropores with diverse diameter and distribution in all trunk regions (Fig. 18) as well as at the female genital orifice and the bursa. The base of the proboscis had sensory pores but no micropores (Fig. 17). These observations were SEM generated and would naturally have been missed by Kostylew [11] and Dinnik [8].

The observed variations in the diameter and distribution of micropores indicate that all trunk regions are involved in the process of absorption of nutrients to various degrees. A few other acanthocephalans species were observed to have a porous tegument surface of the trunk, i.e., micropores, similar to those observed in *Leptorhynchoides. polycristatus* Amin, Heckmann, Halajian, El-Naggar, Tavakol, 2013 and others listed in Amin et al. [4]. Wright and Lumsden [18] and Byram and Fisher [6] further reported that these peripheral canals are continuous, with canalicular crypts. These crypts appear to constitute a huge increase in external surface area implicated in nutrient uptake [6, 18]. Whitfield [17] estimated a 44‐fold increase at a surface density of 15 invaginations per 1 μm^2^ of the tegumental surface of *Moniliformis moniliformis* (Bremser, 1811) Travassos, 1915 (see Byram and Fisher [6]). Surface crypts may also be involved in pinocytosis and lysosomal activity [13].

### Molecular analysis

**Figure 25.**
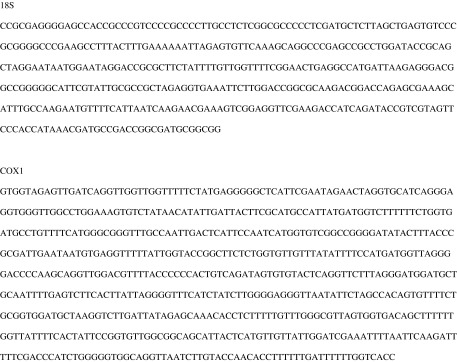
*18S* and *COX1* putative sequences from *E. baeri*. As noted in the text, the sequence data for these specimens was equivocal.

Ninety-two percent (47/51) of the cloned *COX1* sequences and 88% (36/42) of the cloned *18S* sequences were 81% most similar to vertebrates including human (*Homo sapiens*) and brown trout (*S. trutta*) (data not shown). Four of the cloned *COX1* sequences and 6 of the cloned *18S* sequences were most similar to *Echinorhynchus truttae* with 72–81% similarity. Although it is likely that the sequences most closely related to *E. truttae* obtained at low frequency from cloned PCR product sequence are *E. baeri*, the admixture of divergent sequences in the *E. baeri* samples available for molecular analysis in this study makes the determination of *E. baeri* sequence equivocal for the *COX1* and *18S* genes. For further study, we include the *18S* and *COX1* sequences as Figure 25, but are not confident in posting the sequences in GenBank.

## Conclusions

It appears that the three studied populations of *E. baeri* have been diversifying to their present state from a common ancestor that inhabited waters of the IAL drainage system between 10.3 million and 2 million years ago (Middle Miocene-Pliocene). The two populations from Lake Sevan may have diversified more recently. We do not know which of the three studied populations is closer to the ancestral form. However, we have some idea as to the timeline involved in evolutionary changes leading to the degree of diversification evident in each of the three populations studied. Presently, we have no evidence that the intermediate character states of the Turkish population may be closest to those of the ancestral forms from which the two Lake Sevan populations would have diversified. We also have no evidence that E*. baeri* may have been derived from *Echinorhynchus truttae* Schrank, 1788 as speculated by Platonova [16]. Further diversification over even a longer period of time may lead to the evolution of three distinct species, and perhaps more elsewhere in the same drainage system. The above conclusions do not exclude the possibility that the Turkish population may represent a cryptic species. However, we have decided to leave the question of potential cryptic species for future studies.
